# Comprehensive *BRCA1/2* mutation landscape in prostate cancer in the UAE and Arab population

**DOI:** 10.3389/fcell.2026.1737736

**Published:** 2026-05-26

**Authors:** Zainab M. Al Shareef, Rula M. Al-Shahrabi, Poorna Manasa Bhamidimarri, Burcu Yener, Amal Bouzid, Alaa Mohamed Hamad, Shirin Murad, Ahmed Elbarkouky, Fatemeh Saheb Sharif-Askari, Riyad Bendardaf, Rifat A. Hamoudi, Mahmood Y. Hachim

**Affiliations:** 1 Department of Basic Medical Sciences, College of Medicine, University of Sharjah, Sharjah, United Arab Emirates; 2 Research Institute of Medical & Health Sciences, University of Sharjah, Sharjah, United Arab Emirates; 3 Department of Pathology, Al Qassimi Hospital, Sharjah, United Arab Emirates; 4 Department of Pharmacy Practice and Pharmacotherapeutics, College of Pharmacy, University of Sharjah, Sharjah, United Arab Emirates; 5 Oncology Unit, University Hospital of Sharjah, Sharjah, United Arab Emirates; 6 Clinical Sciences Department, College of Medicine, University of Sharjah, Sharjah, United Arab Emirates; 7 Division of Surgery and Interventional Science, University College London, London, United Kingdom; 8 College of Medicine, Mohammed Bin Rashid University of Medicine and Health Sciences, Dubai Healthcare City, Dubai, United Arab Emirates

**Keywords:** BRCA, DNA repair, homozygous, mutations, prostate cancer, UAE

## Abstract

**Background:**

Prostate cancer (PCa) is the most common malignancy among men in the United Arab Emirates (UAE) and is often diagnosed at advanced stages with aggressive features. Germline mutations in DNA-repair genes, especially *BRCA1* and *BRCA2*, increase PCa risk, with *BRCA2* conferring up to 8.6-fold and *BRCA1* a 3.7-fold risk. The objective is to determine the prevalence, zygosity, and clinical significance of *BRCA1/2* mutations in high-grade PCa among UAE and Arab patients and describe their potential role in disease aggressiveness.

**Methods:**

A retrospective analysis was performed on 40 archived formalin-fixed, paraffin-embedded prostate tissues (2011–2022), comprising 23 PCa and 17 benign prostatic hyperplasia (BPH). Targeted exon sequencing was performed. Variants were classified using ACMG/AMP criteria using ClinVar and Varchat. Associations between mutation patterns, zygosity, and tumor grade were evaluated.

**Results:**

*BRCA1* mutations occurred in 47.5% of cases, all in exon 10, with 62.5% homozygosity; most frequent were c.794G>A and c.721G>A. BRCA2 mutations occurred in 55% of cases, mainly exon 11, with 68% homozygosity; c.5917A>C and c.5908T>A were most common. Homozygous mutations enriched in high-grade PCa (Grade Groups 3–5) suggested biallelic inactivation and homologous recombination repair deficiency. Several novel variants clustered in DNA repair domains, including *BRCA1* coiled-coil and *BRCA2* RAD51-binding.

**Conclusion:**

Our findings reveal a high prevalence of homozygous BRCA1/2 mutations, with the majority had aggressive disease phenotypes. Therefore, support the potential utility of PARP inhibitors as molecularly targeted therapeutic alternatives to conventional chemotherapy in mutation-positive patients. Furthermore, the identification of novel population-specific variants underscores the urgent need for ethnicity-informed genetic screening protocols, facilitating earlier detection of hereditary risk and enabling informed treatment stratification in United Arab Emirates and Arab men with PCa.

## Introduction

Prostate cancer (PCa) is a major global health issue, with 1.47 million new cases reported in 2022, and the number is projected to nearly double by 2040 due to aging populations (James et al., 2024). The age-standardized incidence of PCa is highest among predominantly White populations in Northern Europe, Australia, and New Zealand, while mortality rates are greatest among populations of African descent in Southern Africa and the Caribbean (Ferlay et al., 2024). Age, family history, ethnicity, and genetic predisposition are well-established risk factors for PCa (Al Shareef et al., 2024).

In the United Arab Emirates (UAE), PCa has emerged as a significant health burden over the past decade, ranking as the most common cancer in men according to age-specific incidence rates (ASIR) (Ferlay et al., 2024). The rising incidence is likely driven by aging demographics, lifestyle-related factors, and family history (Al Shareef et al., 2024). Alarmingly, the disease is often diagnosed at late stages, frequently presenting with high-grade and metastatic cancer, which complicates treatment and worsens outcomes (Pritchard et al., 2016).

Breast Cancer Gene 1 (*BRCA1*) and Breast Cancer Gene 2 (*BRCA2*) are tumor suppressor genes that preserve genomic stability through DNA repair (Gorodetska et al., 2019). Mutations in these genes markedly increase the risk of developing multiple cancers, including PCa (Gorodetska et al., 2019). Germline DNA-repair gene mutations are strongly linked to higher PCa risk across ethnic groups, with BRCA2 carriers facing up to an 8.6-fold increase and BRCA1 carriers a 3.7-fold increase (Pritchard et al., 2016). *BRCA2* mutations in particular are associated with earlier disease onset, higher T stage, advanced Gleason score, and shorter PCa-specific survival (Ibrahim et al., 2018). These findings highlight the critical need for comprehensive cancer screening and monitoring strategies for *BRCA* carriers.

A key challenge in PCa lies in its marked intra-tumoral and inter-patient heterogeneity, which becomes more evident with aging complicates diagnosis and prognosis (Ramón et al., 2020). In advanced disease, tumor aggressiveness and tumor–microenvironment interactions drive chronic inflammation and systemic complications that often contribute more to mortality than the tumor burden itself (Ramón et al., 2020).

While *BRCA1/2* mutations are well-established drivers of breast and ovarian cancers, their contribution to PCa risk, aggressiveness, and clinical behaviour remains poorly characterized particularly in the UAE and broader Arab region. Existing evidence is derived predominantly from Western cohorts, which limits its generalizability to genetically and ethnically distinct populations. This disparity represents a critical gap in the literature, as Arab men with PCa may harbor unique mutation spectra and zygosity patterns that differ meaningfully from those reported in European or North American studies. Understanding the prevalence and functional significance of *BRCA1/2* mutations in this population is therefore essential for developing ethnicity-informed risk stratification, genetic counselling protocols, and targeted treatment strategies tailored to Arab men with PCa (Safiri et al., 2025).

In this study, we investigated *BRCA*1/2 mutations in Emirati and Arab men with PCa. Our results point to the need for *BRCA* testing as part of standard care to inform treatment choices, enable family counselling, and build a regional variant reference for precision oncology. These efforts complement ongoing national genomic programs designed to improve population health and strengthen global advances in genomic medicine.

## Materials and methods

### Ethical approval

This study was conducted in accordance with the Declaration of Helsinki and was approved by the University Hospital Sharjah Ethics Committee (Ref: UHS-HERC-085-10012022) and Funded by University of Sharjah (Grant No. 22010901122). Informed consent was waived by the institutional ethics committee due to the retrospective study design.

### Study design

A retrospective descriptive analysis was conducted on archived prostate tissue samples collected between 2011 and 2022 from the pathology laboratories of University Hospital Sharjah and Al Qassimi Hospital. A comprehensive sampling strategy was employed, whereby all available samples meeting the inclusion criteria within the study period were included, no formal sample size calculation was performed, as the study was purely descriptive in nature with no inferential statistical comparisons intended. A total of 40 formalin-fixed, paraffin-embedded (FFPE) blocks were selected, including 23 PCa and 17 benign prostatic hyperplasia (BPH) cases. Inclusion criteria were histopathologically confirmed primary diagnosis of PCa or BPH by a certified pathologist, and male adult patients with confirmed prostate pathology. Exclusion criteria were those cases lacking histopathologically confirmed prostate pathology or with inconclusive pathological diagnosis. All FFPE blocks selected for DNA extraction were reviewed by a certified pathologist. The blocks used for molecular analysis were the same blocks from which histopathological diagnosis and Gleason grading were established, confirming the presence of adequate tumoral tissue and formal tumor cellularity percentage threshold. Targeted exon sequencing of the *BRCA1* and *BRCA2* genes was performed to investigate mutational patterns associated with PCa progression.

### DNA extraction

Genomic DNA was extracted from five to six consecutive 5 μm FFPE sections using the QIAamp DNA Tissue Kit (Qiagen, Germany) as per manufacturer’s instructions. Deparaffinization was achieved with xylene followed by ethanol washes. Samples were digested with proteinase K in ATL buffer at 56 °C for 2.5 h. DNA was eluted in 30 µL nuclease-free water and quantified using a NanoDrop 2000 spectrophotometer (Thermo Fisher Scientific, Waltham, MA, USA). A total of 10 ng input DNA was used for library preparation.

### Primer designing and validation

Primers targeting hotspot regions in BRCA1 and BRCA2 were designed using public cancer genomics databases See Supplementary Tables 1, 2 for full BRCA1 and BRCA2 variant details, respectively. Functional domains were identified, and primers were generated via Primer3 and validated with OligoAnalyzer. Sequences and expected amplicon sizes are listed in (Supplementary Tables 3, 4). Primers averaged 20 bp in length, with melting temperatures of 58 °C–62 °C and 47% GC content. Specificity was assessed using the UCSC Genome Browser. Validation on FFPE DNA confirmed amplicon sizes via 2% agarose gel electrophoresis (MBG, Bio Basic Inc.).

### Targeted DNA sequencing using Fluidigm access array

Validated target-specific primers were tagged with Fluidigm-specific sequences and used for targeted next-generation sequencing. Primer mixes were prepared from 100 µM stocks and diluted to 10 µM. Each reaction used 10 ng of FFPE-derived DNA and 10 µM primers with FastStart High Fidelity Master Mix (Roche), under the following conditions: 95 °C for 10 min; two cycles of 95 °C for 15 s, 60 °C for 4 min; and 13 cycles of 95 °C for 15 s, 72 °C for 4 min. Amplicons were purified using ExoSAP-IT and amplified on the Fluidigm 48.48 Access Array IFC as previously described (Presneau et al., 2015). This approach is particularly appropriate for low-input degraded FFPE-derived DNA, as it enables highly multiplexed amplicon-based library preparation from as little as 10 ng of input DNA, ensuring reliable sequencing coverage across targeted *BRCA1* and *BRCA2* exonic regions.

PCR products were harvested, barcoded, and pooled (1 µL/well). Libraries were purified with AMPure XP beads, quantified using a 6% acrylamide gel and Bioanalyzer, diluted to ∼100 pM, and sequenced on the Ion S5 XL system using Ion 520™ Chips and Ion OneTouch2 following the manufacturer’s instructions (Thermo Fisher).

### Variant analyses and interpretation

Sequenced reads were aligned to the reference human genome NCBI 37 (hg19) by Burrows-Wheeler Aligner (BWA) on default settings (Li and Durbin, 2009).

BAM files were generated via Ion Torrent Suite v5.12.3, and reads were visualized using IGV. To ensure analytical sensitivity and specificity, quality thresholds were applied during variant calling: a minimum read depth of >30× per variant position and a variant allele frequency (VAF) ≥80% to confidently classify variants as homozygous. Annotation was based on RefSeq transcripts NM_007294.4 (BRCA1) and NM_000059.4 (*BRCA2*), following HGVS nomenclature (O'Leary et al., 2016). Pathogenicity assessment was conducted by mapping the identified variants to these transcripts and annotating them using ClinVar and Varchat (De Paoli et al., 2024), which incorporates automated scoring based on the ACMG/AMP 2015 classification criteria. Variants were then classified as pathogenic, likely pathogenic, benign, likely benign, or variants of uncertain significance (VUS), or not reported following established interpretation frameworks as previously described (Nykamp et al., 2017).

### Statistical analysis

Descriptive statistics were used to summarize patient demographic and clinical characteristics. Continuous variables, including age and PSA levels, are expressed as median and interquartile range (IQR). Categorical variables, including mutation frequency, zygosity patterns, and tumour grade groups, are expressed as counts and percentages.

## Results

The study cohort consisted of 40 male patients with a median age of 73 (69.75-79.25) years. Among them, 23 individuals (58%) were diagnosed with PCa, while 17 (42%) had BPH. The majority of patients were of Emirati origin (60%), with others from the Eastern Mediterranean (25%) and North African (15%) regions. As expected, prostate-specific antigen (PSA) levels were markedly elevated in PCa cases 23.6 (10.8–88.5) ng/mL compared to BPH cases 4.0 (4.0–5.0) ng/mL, consistent with disease pathology. Histopathological grading of PCa samples, based on International Society of Urological Pathology (ISUP) guidelines and Gleason scoring, revealed a predominance of aggressive tumors. Specifically, 56% of PCa cases (13/23) fell within Grade Groups 3 to 5, including Gleason scores 4 + 3, 8, and 9–10 Table 1.

**TABLE 1:** Baseline demographic and clinical characteristics of the study cohort (n = 40). The table summarizes the distribution of PCa and benign prostatic hyperplasia (BPH) cases, along with the nationality, age, and PSA levels of participants, histopathological characteristics of PCa cases (n = 23), categorized by International Society of Urological Pathology (ISUP) Grade Groups and corresponding Gleason scores. The table presents the number and percentage of patients within each grade group.

Characteristics	No (%)
No. of patients	40
PCa	23 (58)
Benign prostatic hyperplasia	17 (42)
Nationality^a^	No (%)
UAE	24 (60)
^a^Eastern Mediterranean	10 (25)
^a^North African	6 (15)
Age (Median (Q1 – Q3))	73 (69.75–79.25)
PSA level (Median (Q1 – Q3))	
PCa	23.6 (10.8–88.5) ng/mL
Benign prostatic hyperplasia	4.0 (4.0–5.0) ng/mL
Grade (Score), No (%)	
Grade Group 1, Gleason 6	5 (22)
Grade Group 2, Gleason 3 + 4 = 7	2 (9)
Grade Group 3, Gleason 4 + 3 = 7	3 (13)
Grade Group 4, Gleason 8	4 (17)
Grade Group 5, Gleason 9–10	9 (39)

^a^ All patients included in this cohort are of Arab ethnicity and Caucasian race, comprising Emirati nationals (60%), patients of Eastern Mediterranean Arab origin (25%), and patients of North African Arab origin (15%).

Targeted sequencing revealed recurrent mutational events in two genomic regions: chr17:41246704–41246874 in *BRCA1* and chr13:32914233–32914467 in *BRCA2,* prompting focused investigation of these loci, additional details are provided in Supplementary Figure 1.

### *BRCA1* mutations

**FIGURE 1:**
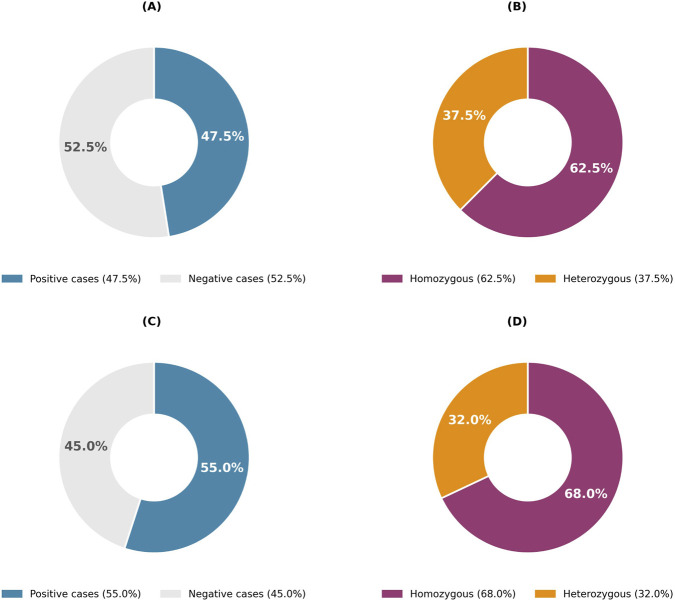
*BRCA1/2* Variant Prevalence and Zygosity Patterns in UAE and Arab Prostate Cancer Cohort. Prevalence of *BRCA1* variants in the total cohort (n = 40); 47.5% of patients (19/40) tested positive, including 12 PCa and 7 BPH cases, with all mutations localized within exon 10. **(B)** Zygosity distribution among *BRCA1*-positive cases (n = 19); homozygous mutations predominated (62.5%) over heterozygous (37.5%), consistent with biallelic inactivation. **(C)** Prevalence of *BRCA2* variants in the total cohort (n = 40); 55.0% of patients (22/40) tested positive, including 8 PCa and 5 BPH cases, with 19 distinct variants localized within exon 11. **(D)** Zygosity distribution among BRCA2-positive cases (n = 22); homozygous mutations predominated (68.0%) over heterozygous (32.0%), with enrichment in 65.5% of high-grade PCa tumors **(A,B,C,D)**.

As shown in Table 2, *BRCA1* variants were detected in 47.5% of the total cohort (19/40), including 12 PCa and 7 BPH cases. All mutations were located within exon 10. Notably, 77% of high-grade PCa cases carrying *BRCA1* mutations exhibited homozygous alterations, suggesting biallelic inactivation. Across all *BRCA1*-positive samples, homozygous mutations were more frequent (62.5%) than heterozygous 37.5%, as illustrated in Figure 1B.

**TABLE 2:** Mutation characteristics and frequency in *BRCA1* gene. *BRCA1* mutations identified in this study were mapped to the reference transcript NM_007294.4 according to NCBI RefSeq database. The corresponding table presents detailed information including genomic coordinates, exon locations, coding sequence changes, predicted pathogenicity, mutation frequencies, associated diagnoses, and zygosity patterns. VUS: variants of uncertain significance.

Exon	Region	Coding sequence (cDNA)	Type	Pathogenicity	Freq (N/40), %	Diagnosis	Zygosity	(Ref Seq)
10	Chr 17:41246741	c.805C>T	Stop codon	Not reported	4 (10)	BPH	(3) Homozygous (1) Heterozygous	Not reported
10	Chr 17:41246514	c.1035T>C	Missense	Benign	1 (2.5)	PCa 8 (4 + 4)	(1) Heterozygous	rs1164807386
10	Chr 17:41246754	c.794G>A	Missense	Not reported	13 (32.5)	BPH	(1) Heterozygous	Not reported
​	​	​	​	​	​	BPH	(2) Homozygous	​
​	​	​	​	​	​	PCa 9 (5 + 4)	(2) Homozygous	​
​	​	​	​	​	​	PCa 9 (4 + 5)	(1) Heterozygous	​
​	​	​	​	​	​	PCa 8 (4 + 4)	(1)Homozygous	​
​	​	​	​	​	​	PCa 7 (4 + 3)	(1) Homozygous	​
​	​	​	​	​	​	PCa 7 (3 + 4)	(1)Heterozygous	​
​	​	​	​	​	​	PCa 6 (3 + 3)	(3) Homozygous	​
​	​	​	​	​	​	PCa 6 (3 + 3)	(1) Heterozygous	​
10	Chr17:41246825	c.721G>A	Missense	Benign	13 (32.5)	BPH	(1) Heterozygous	rs1379167990
​	​	​	​	​	​	BPH	(2) Homozygous	​
​	​	​	​	​	​	PCa 9 (5 + 4)	(2) Homozygous	​
​	​	​	​	​	​	PCa 9 (4 + 5)	(1) Heterozygous	​
​	​	​	​	​	​	PCa 8 (4 + 4)	(1)Homozygous	​
​	​	​	​	​	​	PCa 7 (4 + 3)	(1) Homozygous	​
​	​	​	​	​	​	PCa 7 (3 + 4)	(1)Heterozygous	​
​	​	​	​	​	​	PCa 6 (3 + 3)	(3) Homozygous	​
​	​	​	​	​	​	PCa 6 (3 + 3)	(1) Heterozygous	​
10	Chr 17:41246833	c.717G>A	Missense	Not reported	3 (7.5)	BPH	(1) Heterozygous	Not reported
​	​	​	​	​	​	BPH	(1) Homozygous	​
​	​	​	​	​	​	PCa 7 (4 + 3)	(1) Homozygous	​
10	Chr 17:41246837	C.709T>C	Missense	Not reported	1 (2.5)	BPH	(1) Heterozygous	Not reported
10	Chr 17:41246806	c.744T>C	Silent	VUS	1 (2.5)	BPH	(1) Homozygous	rs879255288
10	Chr 17:41246748	c.800G>T	Stop-loss Mutation	Not reported	2 (5)	PCa 7 (3 + 4)	(2) Heterozygous	Not reported
						PCa 6 (3 + 3)		
10	Chr 17:41246778	c.770T>C	Missense	VUS	1 (2.5)	PCa 6 (3 + 3)	(1) Heterozygous	De Paoli et al. (2024)
10	Chr 17:41246774	c.772T>C	Missense	Not reported	1 (2.5)	PCa 9 (4 + 5)	(1) Homozygous	Not reported

The most frequently observed *BRCA1* variants were c.794G>A and c.721G>A (both missense), each present in 13 patients (32.5%). The c.805C>T (stop codon) variant appeared in 10% of the cohort, and c.717G>A in 7.5%.

According to established databases, c.721G>A is considered benign, while c.794G>A and c.805C>T were not previously reported. Notably, c.794G>A was predominant in PCa cases, whereas c.717G>A was more common among BPH cases.

### *BRCA2* mutations

As shown in Table 3, a total of 19 distinct *BRCA2* variants were identified in exon 11, occurring in 22 samples (55%). These included 8 PCa and 5 BPH cases Table 3. Homozygous mutations were again predominant, present in 68% of *BRCA2*-positive cases and in 65.5% of high-grade PCa tumors as illustrated in Figure 1D.

**TABLE 3:** Mutation characteristics and frequency in *BRCA2* gene. *BRCA2* mutations identified in this study were mapped to the reference transcript NM_000059.4 according to NCBI RefSeq database. Reported data include the genomic coordinate, exon location (exon 11), cDNA position and change, mutation type, predicted pathogenicity observed frequency among samples, diagnosis (grading of PCa cases and/or BPH), zygosity pattern and reference variant identifier (RefSeq or clinvar registered number). VUS: variants of uncertain significance.

Exon	Region	Coding sequence (cDNA)	Type	Pathogenicity	Freq (N/40), %	Diagnosis	Zygosity	Ref Seq
11	Chr13:32914538	c.6046T>C	Missense	Not reported	1 (2.5)	PCa 9 (5 + 4)	Homozygous	rs2137523632
11	Chr13:32914537	c.6043G>A	Silent	likely benign/VUS	1 (2.5)	PCa 9 (4 + 5)	Homozygous	rs1566233782
11	Chr13:32914531	c.6039A>G	Silent	Benign	1 (2.5)	BPH	Heterozygous	rs1593907331
11	Chr13:32914523	c.6033T>A	Missense	Not reported	2 (5)	BPH	(2) Heterozygous	rs2137523475
						PCa 9 (4 + 5)		
11	Chr13:32914520	c.6028G>A	Missense	VUS	1 (2.5)	BPH	Homozygous	rs1555284504
11	Chr13:32914504	c.6012A>G	Silent	Benign	1 (2.5)	PCa 8 (4 + 4)	Heterozygous	rs572976024
11	Chr13:32914480	c.5988A>T	Silent	Benign	1 (2.5)	PCa 9 (4 + 5)	Heterozygous	rs773045221
11	Chr13:32914472	c.5980C>T	Stop codon	Pathogenic	2 (5)	PCa 9 (4 + 5)	Heterozygous	rs80358831
						BPH	Homozygous	
11	Chr13:32914469	c.5977T>C	Silent	Benign	2 (5)	BPH	Heterozygous	RCV001434012
						PCa 8 (4 + 4)	Homozygous	RCV003584944
11	Chr13:32914437	c.5945G>A	Missense	VUS	1 (2.5)	PCa 7 (4 + 3)	Heterozygous	rs28897738
11	Chr13:32914442	c.5952A>G	Silent	Benign	1 (2.5)	BPH	Homozygous	rs786201742
11	Chr13:32914445	c.5953T>C	Missense	Not reported	3 (7.5)	PCa 9 (4 + 5)	(3) Heterozygous	Not reported
		c.5954C>T	Missense	VUS				rs1272409475
		c.5955T>A	Silent	Benign				RCV002157061
11	Chr13:32914452	c.5960A>C	Missense	Not reported	2 (5)	PCa 8 (4 + 4)	Heterozygous	Not reported
	​					BPH	Heterozygous	
11	Chr13:32914422	c.5930T>A	Missense	likely benign/VUS	5 (12.5)	BPH	(2) Heterozygous	rs1566233570
						PCa 7 (3 + 4)	Heterozygous	
						PCa 9 (4 + 5)	Heterozygous	
						PCa 6 (3 + 3)	Heterozygous	
11	Chr13:32914418	c.5927G>T	Missense	VUS	1 (2.5)	PCa 7 (3 + 4)	Heterozygous	rs587782751
11	Chr13:32914400	c.5908T>A	Missense	Not reported or VUS	17 (42.5)	7 BPH 10 PCa 5 (4 + 5), 2 (4 + 4) 1 (3 + 4), 2 (3 + 3)	Homozygous	rs2137522180
11	Chr13:32914409	c.5917A>C	Missense	likely benign/VUS	17 (42.5)	7 BPH 10 PCa 5 (4 + 5), 2 (4 + 4) 1 (3 + 4), 2 (3 + 3)	Homozygous	rs1555284460

**FIGURE 2:**
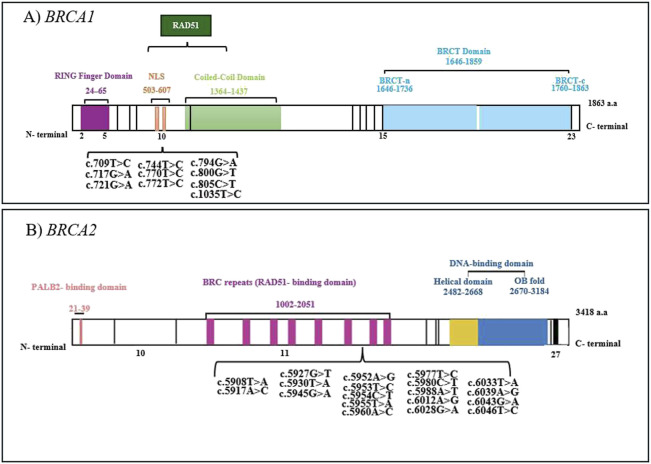
Summary of domain architecture of human *BRCA1* and *BRCA2* with germline variants identified in the current cohort. *BRCA1* **(A)** and *BRCA2* **(B)** gene/protein domains, highlighting exons, domain labelling, and identified mutations.

As detailed in Table 3, the most frequently identified *BRCA2* variants were c.5917A>C and c.5908T>A (missense), each found in 17 patients (42.5%). Other recurrent mutations included c.5930T>A (12.5%), and lower-frequency variants such as c.6033T>A, c.5980C>T (stop codon), c.5977T>C (silent), and c.5960A>C (missense). The c.5980C>T mutation was previously reported as pathogenic; others were either benign, of uncertain significance, or unreported. A cluster of mutations affecting amino acid position chr13:32914445 (c.5953T>C, c.5954C>T, c.5955T>A) was detected in three patients, with mixed pathogenicity classifications. Clinical characteristics of *BRCA* carriers are summarized in (Supplementary Table 4; Supplementary Figure 2). Figure 1 present the patterns of *BRCA1* and *BRCA2* variants and zygosity in PCa and BPH Samples. Figure 2 illustrates the domain structures of *BRCA1* and *BRCA2* and maps the mutations identified in this study. The structural framework of the genes, including domain and exon, was adapted from previously described methods (Narod and Foulkes, 2004; Cardoso et al., 2018).

### *BRCA1/2* mutational profiling and clinical relevance

Homozygous mutations predominated across both loci, with notable enrichment in high-grade PCa tumors, consistent with biallelic tumor suppressor inactivation as a contributor to disease progression. Several variants, including *BRCA1* c.794G>A and c.805C>T, were absent from established databases, expanding the known *BRCA* mutational spectrum and highlighting the need for ethnicity-inclusive genomic studies. These findings support the integration of *BRCA* profiling into the clinical management of PCa in UAE and Arab populations, with direct implications for prognostic stratification and PARP inhibitor eligibility.

## Discussion

To our knowledge, this is the first study in the UAE and broader Arab region to comprehensively profile *BRCA1* and *BRCA2* mutations in PCa, with emphasis on zygosity and tumor grade. The predominance of homozygous *BRCA1/2* mutations in high-grade PCa, is consistent with biallelic inactivation and aggressive disease phenotypes in our cohort. This pattern implies complete loss of tumor suppressor function, consistent with *BRCA*’s known role in homologous recombination repair (HRR), where loss of function leads to impaired double-strand break repair and promotes genomic instability (Nyberg et al., 2022).

## Functional implications of *BRCA1/2* inactivation

Loss of *BRCA1*/2 function impairs DNA double-strand break repair, leading to genomic instability, and disease progression in prostate tumors with defective DNA repair mechanisms (Nyberg et al., 2022). Notably, *BRCA1* mutations in our study clustered within exon 10, especially in regions encoding the coiled-coil domain and nuclear localization signals (NLS). These domains mediate interaction with PALB2, a key HRR partner, and their disruption especially when affecting both NLS regions spanning exons 10 to 11 may result in cytoplasmic mislocalization and compromised HRR function (Pulver et al., 2021). The location of the identified mutations suggests potential functional impairment consistent with previous findings (Helleday, 2011). Such mutations may contribute to HRR deficiency and theoretically sensitize cells to Poly (ADP-ribose) Polymerase (PARP) inhibitors through the mechanism of synthetic lethality, as reported in earlier studies (Helleday, 2011).

While *BRCA1* mutations modestly elevate PCa risk, BRCA2 mutations are associated with a significantly greater oncogenic burden (Nyberg et al., 2022; Messina et al., 2020). In our cohort, 85% of BRCA2 mutation carriers had advanced-stage PCa, compared to 40% of *BRCA1* carriers, aligning with previous studies reporting worse PCa-specific survival among *BRCA2* carriers (Messina et al., 2020; Nyberg et al., 2020a). These findings support the utility of stratifying patients by *BRCA* mutation type for risk assessment and management.

### Mutation hotspots and broader cancer relevance

Specific mutation hotspots in *BRCA1* and *BRCA2* are linked to both triple-negative breast cancer (TNBC) and PCa due to disruption of the HRR pathway (Messina et al., 2020). In *BRCA1*, mutations in the coiled-coil domain (exon 10–11) and BRCT domains (exons 16–24) impair DNA repair and are associated with TNBC and poor-prognosis PCa (Chen et al., 2018). In *BRCA2*, the OCCR (c.3249–c.5681) is linked to TNBC, while the PCa Cluster Region (PCCR) (c.756–c.1000 and >c.7914) is enriched in aggressive PCa (Ruan et al., 2023).

*BRCA2* mutations identified in our study clustered within exon 11, which encodes the RAD51-binding domain, similar to *BRCA1*, a region critical for the HRR process. This observation is in line with previous reports identifying a PCCR within *BRCA2*, encompassing the oligonucleotide/oligosaccharide-binding (OB1/OB2) and Tower domains (Nyberg et al., 2020b).

### Novel and known variants suggest population-specific patterns

Although some of the mutations identified in our study are novel, others have been previously reported in the literature as benign or of uncertain significance in the context of breast cancer (Dines et al., 2020). Notably, several of these variants overlap with those implicated in hereditary breast and ovarian cancer syndrome (HBOC) (Crawford et al., 2017). Previous large, population-based, family database study suggested a strong link to early-onset PCa (Beebe-Dimmer et al., 2020). However, the relevance of these specific mutations in PCa remains unclear, as the mutational landscape in PCa often involves different regions of *BRCA1*/2 and other genes such as *ATM, CHEK2*, and *HOXB13* (Kazik, 2024).

Cortesi et al. (2021) evaluated *BRCA* mutation rates of PCa in families with breast and/or ovarian cancers. The study found that 61% of *BRCA1* mutations were in exon 10, while 44% of *BRCA2* mutations were clustered within the PCCR near the 3′terminal region beyond codon 7914 (Cortesi et al., 2021).

Previous regional studies have identified the *BRCA1* c.798_799delTT mutation in exon 10 as a prevalent founder mutation among North African breast cancer patients, accounting for approximately 22% of all *BRCA1*-positive cases (Laraqui et al., 2015). This supports our finding of a mutations clustering within exon 10, specifically between c.700–800 aligning with the location of the c.798_799delTT founder mutation reported in North African populations.

In the case of *BRCA2*, Nyberg et al. (2020) defined the PCCR as spanning from c.6372 or c.6493 to the 3′end of the gene (Nyberg et al., 2020a). Notably, our identified mutations fall within the exonic regions covered by this hotspot. Although *BRCA2* mutations both within and outside the PCCR have been associated with an increased risk of PCa. The same study suggested that carriers of mutations within the PCCR may be at comparatively lower risk than those with mutations outside this region. This pattern mirrors the findings reported in the UK study, which similarly demonstrated an elevated risk associated with PCCR mutations, reporting a hazard ratio of 2.92 (95% CI: 1.54–5.54) for PCCR versus non-PCCR mutations (Nyberg et al., 2020a; Nyberg et al., 2020b; Moran et al., 2012).

### Regional BRCA mutation patterns in Arab and Middle Eastern populations

The mutational spectrum of *BRCA* genes in Arab and Middle Eastern populations with PCa remains insufficiently characterized in the current literature. A study from Morocco reported a combined *BRCA1*/2 mutation frequency of 13.3% (4 out of 30 patients). Notably, 20% of PCa cases occur in men with a family history of the disease, suggesting a potential hereditary component in a subset of cases. The Moroccan cohort identified a *BRCA1* frameshift deletion (c.1956delGAAA, exon 10) and *BRCA2* mutations including a frameshift insertion (c.7235insG; exon 14) and a whole exon 12 deletion (Salmi et al., 2021). More recently, a study from the UAE investigated *BRCA1/2* variants across multiple cancer types through family-based screening. Among the 19 PCa cases, three patients (15.8%) were identified as *BRCA* mutation carriers, corresponding to a 10.5% mutation rate in *BRCA2* (variants c.2254_2257del and c.7558C>T, located in exons 10 and 15, respectively) and a 5.3% mutation rate in *BRCA1* (variant c.1771A>G, located in exon 11) (Al et al., 2025).

In contrast, the present cohort demonstrates a substantially higher mutation prevalence 47.5% for *BRCA1* and 55% for *BRCA2* alongside a distinct mutational signature characterized by recurrent missense variants (c.794G>A, c.5917A>C) and a striking predominance of homozygous alterations absent from both prior regional studies, collectively suggesting population-specific mutational enrichment unique to this Emirati and Arab cohort. Importantly, the recurrence of *BRCA1* mutations in exon 10, *BRCA2* in exon 11 observed in both studies (Salmi et al., 2021; Al et al., 2025) underscores its potential relevance in the genetic landscape of PCa in this region, supporting the need for targeted screening strategies.

### Germline screening and clinical implications

Germline-based PCa screening in *BRCA* mutation carriers is robustly supported by multiple independent studies from dedicated surveillance programs (Fasulo et al., 2025; Fasulo et al., 2023; Lazzeri et al., 2023; Fasulo et al., 2022). Fasulo et al. (2025) demonstrated that genotype-guided PSA screening incorporating the Prostate Health Index (PHI) in unaffected men harboring germline DNA repair pathogenic variants yields significantly superior early detection of clinically significant PCa compared to standard population screening, with high adherence rates among high-risk individuals confirming the feasibility and effectiveness of targeted surveillance protocols in this molecularly defined population (Fasulo et al., 2025). Critically however, Fasulo et al. (2023) documented that male relatives of confirmed germline variant carriers exhibit a profound deficit in awareness of their inherited susceptibility, representing a systemic failure of cascade testing that translates directly into delayed diagnosis, forfeiture of curative intervention, and denial of PARP inhibitor eligibility (Fasulo et al., 2023). Male carrier status is often only identified when a female relative undergoes genetic screening a systemic gap highlighted by Lazzeri et al. (2023), who noted that men remain unaware of their inherited risk until a woman in the family is tested (Lazzeri et al., 2023). Fasulo et al. (2022) further established that the IMPACT screening model should serve as an operational blueprint for extending genotype-guided surveillance to all germline DNA repair gene carriers including *BRCA1/2*, as inherited genomic instability constitutes a unifying high-risk state warranting proactive clinical intervention (Fasulo et al., 2022). In the context of our cohort, where biallelic *BRCA1/2* mutations predominate at strikingly elevated prevalence within a UAE and Arab population already underserved by genetic infrastructure, these converging findings collectively mandate the urgent establishment of germline-first testing pathways, PHI-integrated surveillance protocols, and male-inclusive cascade screening programs as foundational pillars of hereditary PCa management in the Arab world.

### Study limitations and future directions

The study is limited by small sample size. Larger, prospective studies with functional validation are required to confirm pathogenicity, strengthen genotype–phenotype associations, and enable integration of *BRCA* testing into personalized oncology and routine care in Middle Eastern populations.

## Conclusion

Our findings show a high frequency of homozygous *BRCA*1/2 mutations in high-grade PCa within an Arab cohort, particularly in younger men, suggesting a hereditary contribution. Novel variants enriched in critical DNA repair domains (RAD51-binding, OB-fold) highlight their role in genomic instability and aggressive tumor biology. The clinical significance of these findings is substantial. *BRCA2* mutations, in particular, are established predictive biomarkers for PARP inhibitor therapy including olaparib, now incorporated into international guidelines for metastatic castration resistant PCa. The high mutation burden identified here strongly advocates for routine *BRCA* profiling within UAE and Arab oncological practice, enabling timely identification of candidates eligible for targeted therapeutic intervention. This work, in a genetically understudied population, supports the value of population-specific *BRCA* testing with zygosity assessment and domain-level annotation to guide management.

## Data Availability

The datasets presented in this study can be found in online repositories. The names of the repository/repositories and accession number(s) can be found below: NCBI BioProject database under accession number PRJNA1359592 (https://www.ncbi.nlm.nih.gov/bioproject/1359592).
