# Supplementary material for: Comprehensive BRCA1/2 mutation landscape in prostate cancer in the UAE and Arab population
Source: Front Cell Dev Biol. 2026 May 26;14:1737736. doi: 10.3389/fcell.2026.1737736 (PMC13246480; doi:10.3389/fcell.2026.1737736)

Supplementary Material

**Supplementary Figure (1)**

**Supplementary Figure 1:** Schematic representation of the BRCA mutations in patients with prostate cancer (PCa) and benign prostatic hyperplasia (BPH).

The figure categorizes patients with PCa and BPH based on mutation distribution and zygosity (heterozygous, homozygous, or no mutation). The percentages reflect the prevalence in the study cohort.


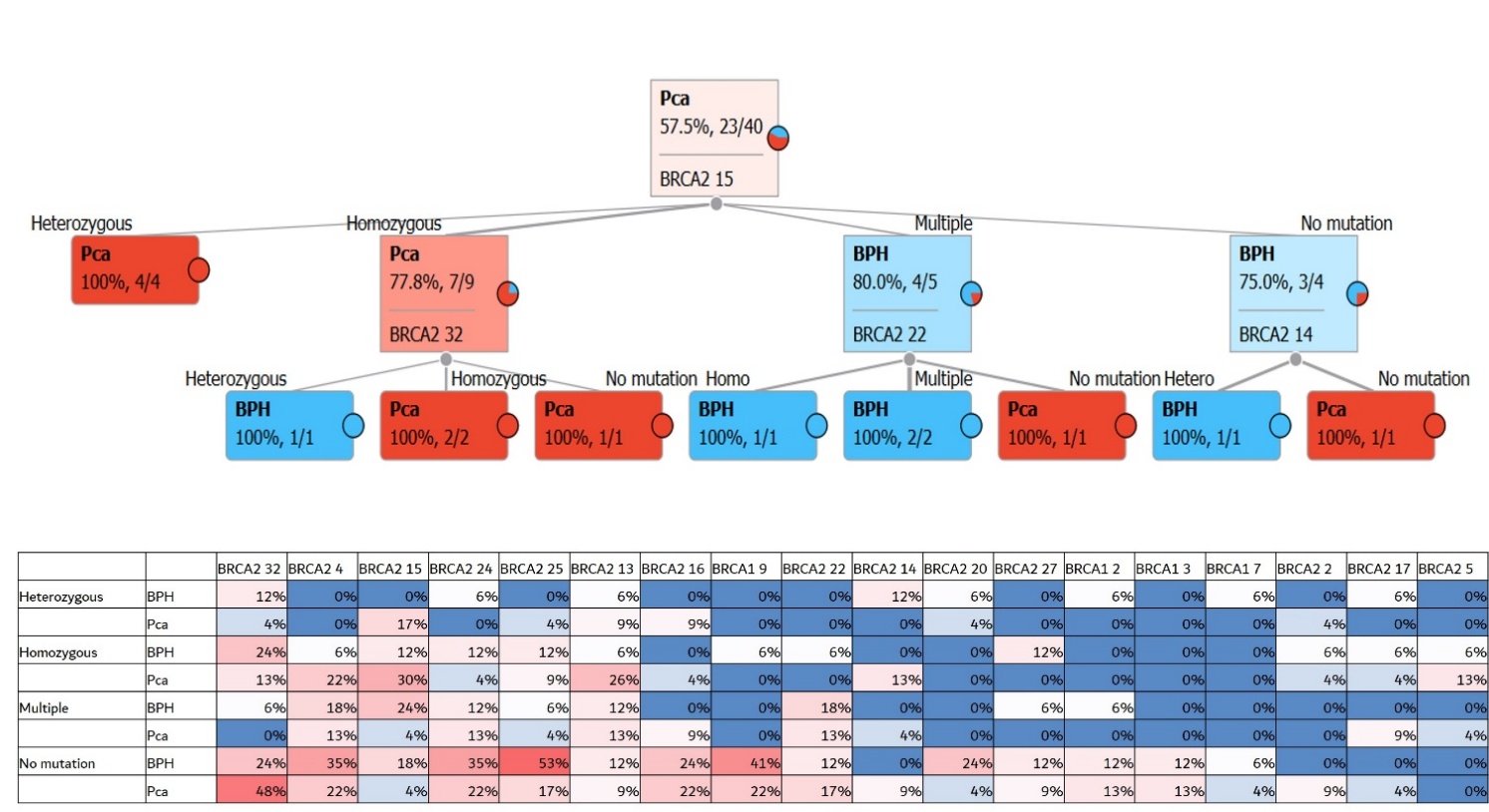

Supplement: Supplementary file 2 [file DataSheet1.docx]
